# Tic Douloureux of Twelve Months’ Standing Cured by Resection of the Infra-Orbital Nerve and Its Branches

**Published:** 1880-12-20

**Authors:** R. S. Jenkins

**Affiliations:** Georgia


### TIC DOULOUREUX OF TWELVE MONTHS STANDING CURED BY RESECTION OF THE INFRA-ORBITAL NERVE AND ITS BRANCHES.

BY R. S. JENKINS, M.D., OF GEORGIA.

Francis Grant, colored, aged fifty-two years, was attacked with inflammatory neuralgia of the left facial nerve, August 28th, 1879. Gave sulph. quinine and sulph. morphia, local blisters, warm poultices, without any amelioration of symptoms except to partially subdue the general inflammation. The neuroses ceased to be general and concentrated itself in the infraorbital and its branches.

August 30th. Was attacked with spasms of the muscles supplied by this nerve and its branches, coming on regularly, lasting from one to three minutes, with from five to eight minutes intermission, and continuing day and night up to the time of the operation.

In a short time after these attacks commenced the patient began to lose her appetite, subsequently becoming very much emaciated; but, under the use of cod-liver oil and iron, the appetite returned, and she gained some flesh, retaining both up to the time of the operation. The neuralgic attacks continued, notwithstanding she had taken ext. of belladonna, quinine, bromide of potassium and various other nervines, without lessening the duration, or increasing the length of time between the attacks.

The spasms could be produced by slight pressure on the nerve, or where the canine tooth should have been, even though an attack had subsided. She had all the teeth extracted from that side, supposing them to be partially the cause of the trouble, without any improvement whatever; also thinking all the time, as she expressed it, that she had been "tricked" by some one of her enemies, and continued to believe thus up to the time of the resection.

August 10th, 1880. Assisted by Drs. Hudson, Boozer and Jones, anæsthetized the patient and performed neurotomy, by making an incision about one and a half inches in length down to the nerve, beginning at the superior portion of the ala of the nose, extending towards the corner of the mouth, dividing the main trunk of the nerve at its exit from the foramen, tracing it for one inch with five branches, and excised them, all of which were enlarged. There was considerable thickening of the mucous membrane of the upper lip, the cheek and conjunctiva of the lower lid of that side of the face, which had subsided to some extent since the operation.

The wound healed by first intention. The patient has taken since half ounce of mur. tinct. iron and five drachms of bromide of potassium.

For several days after the operation, the patient complained of a tingling sensation at the region of the infraorbital foramen, which has now ceased, and up to this time there has been no recurrence of the neuralgic spasm, and the patient is delighted with my having "cut the 'trick' out."

